# Waist-to-height ratio index for predicting incidences of hypertension: the ARIRANG study

**DOI:** 10.1186/s12889-018-5662-8

**Published:** 2018-06-19

**Authors:** Jung Ran Choi, Sang Baek Koh, Eunhee Choi

**Affiliations:** 1 Institute of Genomic Cohort, Yonsei University Wonju College of Medicine, Wonju, Republic of Korea; 2 Department of Preventive Medicine, Yonsei University Wonju College of Medicine, Wonju, Republic of Korea; 3 Institute of Lifestyle Medicine, Yonsei University Wonju College of Medicine, Wonju, Republic of Korea

**Keywords:** Waist-to-height ratio, Hypertension, Community-based prospective study, Predictor, Korean adults

## Abstract

**Background:**

Several anthropometric indices such as body mass index (BMI) and waist circumference (WC) have been examined as indicators of cardiovascular diseases, in both adults and children. However, the waist-to-height ratio (WHtR) is considered a better predictor for the detection of cardiovascular risk factors, than BMI. We investigated the association between the WHtR and incident hypertension.

**Methods:**

A total of 1718 participants, aged 39–72 years, were recruited in this longitudinal study. Participants were divided into 2 groups according to the development of hypertension during 2005–2008 (baseline) and 2008–2011 (follow-up). Logistic regression models were used to evaluate the WHtR as a significant predictor of hypertension.

**Results:**

During the 2.8 years of follow-up, 185 new cases of hypertension (10.8%) were diagnosed, with an incidence rate of approximately 4% per year. The WHtR was significantly higher in the participants who had developed hypertension than in those who had not (0.54 ± 0.05 vs. 0.51 ± 0.05, *p* < 0.001). After adjusting for age, sex, smoking status, alcohol intake, regular exercise status, total cholesterol, and systolic blood pressure, at the baseline, the logistic regression analysis indicated that the participants with the highest quartile of the WHtR (WHtR≥0.54) were 4.51 times more likely to have hypertension than those with the lowest quartile (odds ratio 4.51; 95% confidence interval 2.41–8.43; *p* < .0001). The area under the curve for the WHtR, in identifying hypertension risk, was significantly greater than that for the BMI (*p* = 0.0233).

**Conclusion:**

A positive association between WHtR and the incidence of hypertension was observed in Korean adults. The findings of the present community-based prospective study suggest that the WHtR may be a better predictor of incident hypertension.

## Background

Owing to the increases in the incidences of obesity and lifestyle changes, the prevalence of cardiovascular diseases (CVDs), such as hypertension and diabetes, has been on the rise. It has been suggested that the prevention and regulation of hypertension and diabetes could substantially decrease the risk of CVD development [1, 2]. Hypertension is a one of the risk factors for obesity-related CVDs [3]. Several epidemiological reports have demonstrated an upward trend in the link between the prevalence of increased blood pressure or hypertension and obesity [3–5].

Recently conducted cross-sectional studies have investigated the association of waist circumference (WC), waist-to-hip ratio (WHR), waist-to-height ratio (WHtR), and body mass index (BMI) with the prevalence of hypertension [2, 6–8]. However, in those studies, most of the anthropometric indices, such as BMI and WC, were used for the prediction of obesity-related outcomes such as central obesity, and were not constant between the genders and various ethnic groups [2, 3, 6–8]. In Japan, various cross-sectional studies have demonstrated a stronger association of WHtR with cardiovascular risk factors, compared to BMI and WC [2, 6, 9].

WHtR is a simple and practical tool used for the measurement of metabolic risk factors [1, 10, 11]. A systematic review inferred that WHtR may be beneficial, regardless of the age-, gender-, and ethnicity-specific values [12]. Studies have shown anthropometric indicators can predict hypertension in adolescents [11]. Additionally, it has been observed that obese children with a higher WHtR are more likely to have a higher cardiometabolic risk, and a single WHtR cut-off of 0.5 seems to be suitable for use in children as well as in adults, without age-related distinctions [1, 13, 14]. Recent reports investigated a WHtR cut-off value ≥0.5 and confirmed that high adiposity was strongly connected with cardiovascular disease [9, 10, 15–17]. However, those cut-offs were established in Asian populations; in non-Asian populations, this association may be different [18, 19].

Nevertheless, only a few longitudinal studies have compared the appearance of anthropometric parameters in population-based cohorts [2, 4]. Therefore, it is not yet clear which anthropometric index has the strongest association with hypertension, in Asian populations. To further investigate which of the aforementioned indices is the best predictor of the risk of hypertension [20], we examined the population-specific association between WHtR and the incidence of hypertension, in this Korean prospective cohort study.

## Methods

### Participants

This study was conducted from the Korean Genome and Epidemiology Study on Atherosclerosis Risk of Rural Areas in the Korean General Population (KoGES-ARIRANG), a prospective cohort study to observe the prevalence, incidence and risk factors for chronic degenerative disorders. KoGES-ARIRANG included all adults who dwelled in rural areas of Wonju and Pyengchang, Gangwon-do in South Korea where demographic shifts are infrequent and the population can be followed long-term.

**Fig. 1:**
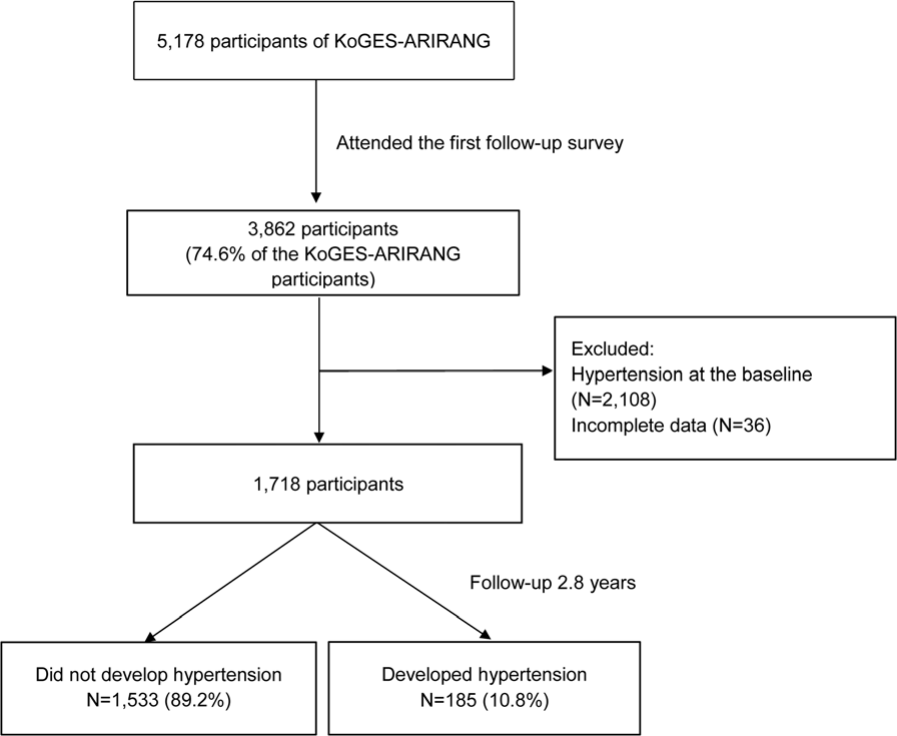
Flow chart of the participants in the KoGES-ARIRANG study

The baseline survey performed from November 2005 to January 2008, contained 5178 adults (2127 men and 3051 women) aged 40 to 70 years. All study subjects were participated in the first follow-up survey (April 2008 to January 2011), of whom 3862 (74.6%) attended. The initial dataset consisted of 3862 participants, aged 39–72 years, on whom the follow-up survey, spanning 2.8 years, was performed. Of these participants, 2144 were excluded due to the presence of hypertension and missing data, especially data on WC and blood pressure. The final dataset comprised 1718 non-hypertensive participants, with a mean age of 53.53 years (Fig. 1). The participants were divided into 2 groups, according to the development of hypertension during 2005–2008 (baseline) and 2008–2011 (follow-up).

This study was approved by the Institutional Review Board of the Wonju Christian Hospital, according to the Helsinki Declaration. All the participants provided informed written consent. Hypertension was defined as a SBP ≥ 140 mmHg, and/or DBP ≥ 90 mmHg, and/or current treatment with antihypertensive medications, at the baseline and follow-up surveys. All the participants were examined after fasting.

### Anthropometric and laboratory measurements

Baseline anthropometric indices such as the BMI and WC, and several parameters were measured. In addition, lifestyle-related factors such as smoking, alcohol intake, and regular exercise status were also investigated. Height and weight were measured twice and then averaged. The WHtR was calculated as the WC (cm) divided by the height (cm). The BMI was calculated by dividing the weight (kg) by the square of the height (m). The WC was measured in the horizontal plane at the mid-point between the anterior iliac crest and the inferior margin of the rib, using a tape measure (SECA-200, SECA, Hamburg, Germany).

Systolic blood pressure (SBP) and diastolic blood pressure (DBP) were measured twice using a standardized mercury sphygmomanometer (Baumanometer, Copiague, NY), on the right arm, and then averaged.

Blood from the participants was extracted into a plain, siliconized glass tube, and serum was separated. Fasting glucose was measured using the hexokinase method. The serum concentrations of low-density lipoprotein (LDL) cholesterol, high-density lipoprotein (HDL) cholesterol, and triglycerides (TGs) were determined using the enzymatic calorimetric method (Advia 1650, Siemens, Tarrytown, NY).

Alcohol and smoking habits were estimated using self-questionnaires. Individuals who had smoked ≥100 cigarettes in their lifetime were defined as current smokers, and those who had not smoked for ≥3 months were defined as ex-smokers. An interview was performed to confirm the use of medications for hypertension, and the status of regular physical exercise.

### Statistical analysis

All the data were analyzed using SPSS version 21.0 (SPSS Inc., Chicago, IL, USA). The baseline characteristics were examined using the chi-square test. To estimate which of the biomarkers was the best predictor of hypertension, we calculated the sensitivity, specificity and mean area under the receiver operator characteristics curve (AU-ROC) of the baseline BMI, WHR and WHtR, and their 95% confidence intervals (CIs), using receiver operating characteristic curves in the group in which hypertension had newly developed. The population-specific risk of hypertension was compared among the quartiles of WHtR. The odds ratios (ORs) of hypertension and their 95% CIs were calculated with reference to the first quartile of each of the measurements, using the logistic regression model. We adjusted for age, gender, alcohol consumption (current), smoking status (current), and physical exercise (yes or no). Additionally, we also adjusted for the baseline SBP, for the hypertension analysis, and the baseline total cholesterol (TC). *P* < 0.05 was considered statistically significant.

## Results

### Baseline characteristics

A total of 1718 participants were included in this study. During the average 2.8-year follow-up, 185 participants (10.8%) developed hypertension. Table 1 shows the mean ± SD, and proportion of the risk characteristics and anthropometric parameters in the participants, for the prediction of the risk of hypertension. The mean WHtR values were higher in the participants in whom hypertension development was observed than in those who did not develop hypertension (0.54 ± 0.05 vs. 0.51 ± 0.05, *p* < .0001). In the participants who developed hypertension, the baseline SBP and TC were 122.1 ± 9.94 mg/dl and 204.30 ± 34.83 mg/dl, respectively, and these parameters were higher than in those who did not develop hypertension (*p* < .0001 vs. *p* = 0.0070). There were no differences in the mean proportions, in terms of smoking habits and the regular exercise status, between the 2 groups (Table 1).

**Table 1:** Baseline characteristics of the study populations, according to the hypertension status at the follow-up

	Did not develop hypertension	Developed Hypertension	*p*
N (%)	1533 (89.2)	185 (10.8)	
Age (yr)	53.12 ± 8.11	56.87 ± 8.01	<.0001
Gender (M)	546 (35.62%)	84 (45.41%)	0.0091
Waist-to-height ratio	0.51 ± 0.05	0.54 ± 0.05	<.0001
Waist-to-hip ratio	0.86 ± 0.07	0.89 ± 0.05	<.0001
Weight (kg)	59.92 ± 9.25	63.37 ± 9.50	<.0001
BMI (kg/m^2^)	23.72 ± 2.90	24.93 ± 2.76	<.0001
SBP (mmHg)	117.17 ± 11.03	122.11 ± 9.94	<.0001
DBP (mmHg)	73.78 ± 7.41	75.54 ± 6.66	0.0021
TC (mg/dl)	196.80 ± 35.81	204.30 ± 34.83	0.0070
TG (mg/dl)	127.48 ± 83.90	142.52 ± 85.12	0.0216
HDL (mg/dl)	46.67 ± 10.81	45.92 ± 10.87	0.3737
LDL (mg/dl)	115.18 ± 30.98	121.71 ± 28.26	0.0063
Fasting glucose (mg/dl)	93.08 ± 19.03	94.49 ± 13.87	0.2127
Current smokers, n (%)	400 (26.13%)	57 (31.15%)	0.1466
Current drinkers, n (%)	617 (40.38%)	96 (52.46%)	0.0017
Regular exercise, n (%)	1074 (70.29%)	144 (78.26%)	0.0241

*BMI* body mass index, *SBP* systolic blood pressure, *DBP* diastolic blood pressure, *TC* total cholesterol, *TG* triglyceride, *HDL* high-density lipoprotein, *LDL* low-density lipoprotein

### Odds ratio for the development of hypertension, according to the WHtR

After adjusting for age, gender, smoking status, alcohol intake, regular exercise, baseline TC, and SBP, the OR of the WHtR was found to be significantly associated with a 4.5-fold increase in the risk of hypertension. A higher WHtR, at the baseline, was positively and significantly associated, in a ratio-dependent manner, with the development of hypertension, in both the crude and adjusted conditional logistic regression models. The OR of hypertension for the highest vs. lowest quartile of WHtR was statistically significant (OR 4.51; 95% CI 2.41–8.43); *p* < .0001) (Table 2).

**Table 2:** Odds ratio of new-onset hypertension, according to the waist-to-height ratio

	Quartile 1	Quartile 2	Quartile 3	Quartile 4	*p* value
WHtR	< 0.4752	0.4752–0.5083	0.5083–0.5440	≥0.5440	
No. of participants	432	427	430	429	
Incidence	13 (3.01%)	39 (9.13%)	60 (13.95%)	73 (17.02%)	<.0001
Crude OR	1	3.24 (1.70–6.16)	5.23 (2.82–9.67)	6.61 (3.60–12.12)	<.0001
Model 1	1	2.99 (1.57–5.71)	4.47 (2.41–8.32)	5.51 (2.98–10.19)	<.0001
Model 2	1	2.95 (1.54–5.63)	4.36 (2.34–8.13)	5.00 (2.69–9.30)	<.0001
Model 3	1	2.89 (1.51–5.55)	4.04 (2.16–7.57)	4.51 (2.41–8.43)	<.0001

Results are described as odds ratios and 95% confidence intervals. Model 1 was adjusted for age and gender; Model 2 was adjusted for age, gender, smoking status, alcohol intake, and regular exercise; Model 3 was adjusted for age, gender, smoking status, alcohol intake, regular exercise, SBP, and total cholesterol at the baseline

### Comparison of the anthropometric indices for predicting the development of hypertension

**Fig. 2:**
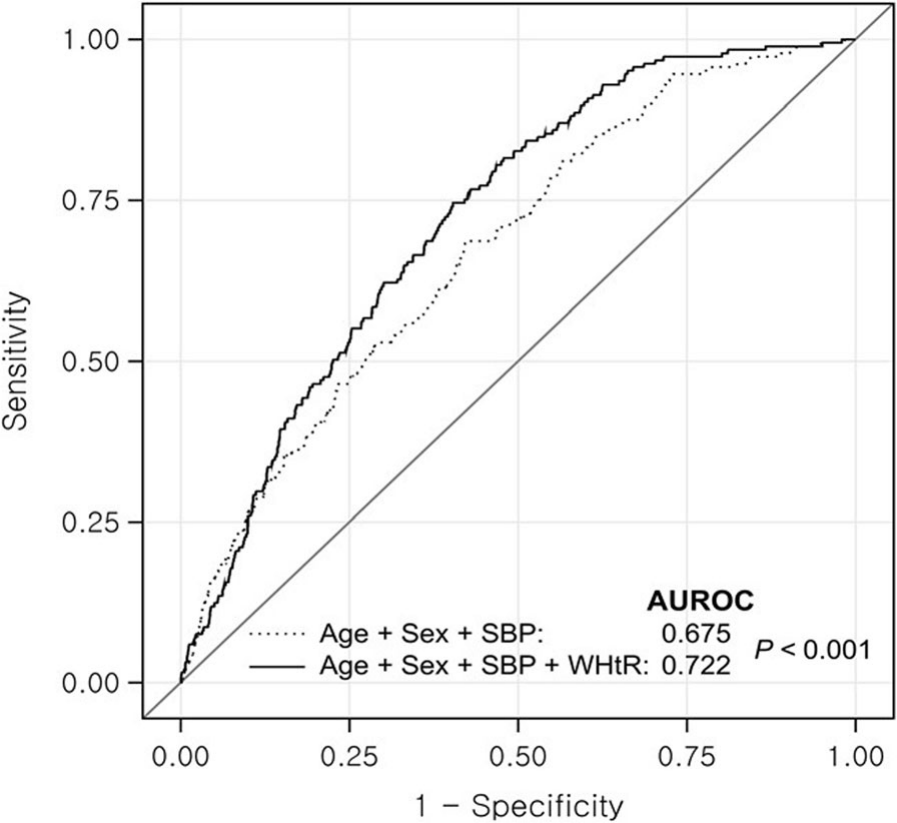
Adjunctive contribution of WHtR to the development of hypertension beyond the information provided by classic risk factors

The mean AU-ROC of the WHtR was higher than that of the BMI (AUC = 0.662; 95% CI, 0.625~ 0.700, *p* = 0.0233) (Table 3). Table 3 presents the AUC of each variable, for the presence of hypertension in Korean adults. According to the respective ROC curves, the WHtR had the highest AUC (AUC = 0.662), followed by the WHR (AUC = 0.648), BMI (AUC = 0.623), and weight (AUC = 0.609). Figure 2 shows the ROC curves for the WHtR added SBP to predict the presence of hypertension in Korean adults.

**Table 3:** Receiver operating characteristic curve analysis for identifying multiple risk factors

	AUC (95% CI)	*p*
Weight (kg)	0.609 (0.567~ 0.651)	–
BMI	0.623 (0.582~ 0.664)	0.3829
WHR	0.648 (0.608~ 0.688)	0.0803
WHtR	0.662 (0.625~ 0.700)	0.0233

*BMI* body mass index, *WHtR* waist-to-height ratio, *AUC* area under the curve, *CI* confidence interval

## Discussion

In the present study, an increased baseline WHtR was observed to be positively associated with the development of hypertension, over a median follow-up period of 2.8 years. The OR of the WHtR was 4.51 (95% CI, 2.41–8.43; *p* < .0001) after adjusting for age, gender, smoking status, alcohol intake, regular exercise status, SBP, and total cholesterol. The mean AU-ROC of the WHtR was higher than that of the BMI (AUC = 0.662; 95% CI, 0.625~ 0.700, *p* = 0.0233).

Diverse anthropometric indices have been developed to identify the risk factors of CVDs, especially hypertension, diabetes, and obesity. However, till date, no definitive measurement tools or indices have been developed for the prediction of hypertension. The most conventionally perceived anthropometric index is the BMI, owing to its simplicity [21, 22]. However, the BMI cannot differentiate between individuals with excess fat and those with a high muscle mass, and cannot discriminate the location of fat; thus, based on the BMI alone, such individuals would be perceived as having the same risk for the development of CVDs [10, 23]. WC is strongly associated with visceral fat depots [24] and is now widely used in the measurement of abdominal obesity [21]. However, WC has a critical point in its use owing to its high collinearity with weight and BMI; thus, its role in the prediction of mortality and health risks is limited [21]. In particular, WC does not take into consideration differences in height. Several studies have demonstrated that individuals with the same WC but different heights are unlikely to have the same cardiometabolic risks [10]. The early detection of hypertension is crucial for its control and prevention, in adults; however, since BP is not routinely tested in most adolescents, it is difficult to monitor the condition in them [11, 25]. Recently, the WHtR was shown to be the most important predictor of BP and hypertension, in Saudi Arabian adult populations [26]. Many studies have also focused on the changes in the values of anthropometric indices such as BMI and WC between men and women, adults and children, and Asian and non-Asian populations [10, 12, 27]. In contrast, WHtR values have been demonstrated to be steady, across age groups, gender, and ethnicities, and are even easier to measure than BMI [12, 27].

Previously conducted cross-sectional studies showed that BMI, WC, and WHR or WHtR were equally associated with the prevalence of hypertension in both genders; however, in other reports, WC was demonstrated to be the single best predictor of hypertension [2, 8, 28–30]. Various prospective studies revealed the association of BMI, a measure of overall obesity, and hypertension [31, 32], while other follow-up studies investigated the WC, WHtR, and WHR, measurements in the case of abdominal adiposity, and found them to be significantly associated with the risk of hypertension [3]. A systematic review and meta-analysis demonstrated that WHtR was a better biomarker for hypertension and CVD risk in both genders, across nationalities and ethnic groups [10, 12]. Like in the case of the aforementioned studies, in the present study too the WHtR was found to be a significant indicator for the incidence of hypertension. However, there are still no absolute measuring tools or indices for the prediction of the incidences of hypertension, regardless of age, gender, and ethnicity, and limited data have been obtained from the prospective studies which focused on the incremental predictive value of the WHtR for the onset of hypertension. In our study, an increasing WHtR was found to be correlated with a consistent increase in the onset of hypertension. Therefore, it is suggested that the WHtR may be a better predicting biomarker for the incidence of hypertension than BMI, and may play a major role in the future diagnosis of hypertension in Korean adults.

Recently, the recommended WHtR value for the prediction of diabetes, cardiovascular disease, hypertension, and metabolic syndrome was set at 0.5. In non-overweight Korean adolescents, a higher prevalence of multiple cardiometabolic risk factors and metabolic syndrome was observed in the WHtR≥0.5 group than the WHtR< 0.5 group [8]. In addition, while the cut-off value for diabetes was estimated to be 0.52–0.53, the corresponding value for hypertension was found to be ≥0.5; these findings are consistent with those of previously conducted studies (data not shown). The mean AU-ROC of the WHtR was higher than that of the BMI (AUC = 0.662; 95% CI, 0.625~ 0.700, *p* = 0.0233). (Table 3). In this study, we measured the anthropometric indices and the diverse parameters that affect the metabolic health status, such as baseline fasting glucose and lipid profiles, in 1718 healthy Korean adults. We then evaluated the development of hypertension after 2.8 years of follow-up.

This study had some limitations. First, the number of participants who developed hypertension after 2.8 years of follow-up was relatively small. Second, the study population was limited to adults from rural areas, and, thus, does not represent the overall Korean population. Third, selection bias could have been present as our study was retrospective in nature.

## Conclusion

The findings of our study suggest that the baseline WHtR is a strong predictor of hypertension in Korean populations. In addition, the WHtR was found to have several advantages as a screening measurement tool, compared to BMI. Intensive lifestyle modifications should be introduced early to reduce the WC to less than half of the height in order to reduce the risk of hypertension. Future studies conducted on large, diverse populations are required to validate the clinical applications of the WHtR.
